# Analysis of QTL for Grain Size in a Rice Chromosome Segment Substitution Line Z1392 with Long Grains and Fine Mapping of *qGL-6*

**DOI:** 10.1186/s12284-020-00399-z

**Published:** 2020-06-11

**Authors:** Ting Zhang, Shiming Wang, Shuangfei Sun, Yi Zhang, Juan Li, Jing You, Tian Su, Wenbo Chen, Yinghua Ling, Guanghua He, Fangming Zhao

**Affiliations:** Rice Research Institute, Key Laboratory of Application and Safety Control of Genetically Modified Crops, Academy of Agricultural Sciences, Southwest University, Chongqing, 400715 China

**Keywords:** Rice, Chromosome segment substitution line, Grain length, QTL mapping, Epistatic analysis

## Abstract

**Background:**

Grain size affects not only rice yield but is also an important element in quality of appearance. However, the mechanism for inheritance of grain size is unclear.

**Results:**

A rice chromosome segment substitution line Z1392, which harbors three substitution segments and produces grains of increased length, was identified. The three chromosome segments were located on chromosomes 1, 5, and 6, and the average length of the substitution segment was 3.17 Mb. Cytological analysis indicates that the predominant cause of increased grain length in Z1392 could be cell expansion in the glumes. Seven quantitative trait loci (QTLs) for grain size related traits were identified using the secondary F_2_ population produced by Nipponbare/Z1392. The inheritance of grain length in Z1392 was mainly controlled by two major QTLs, *qGL-5* and *qGL-6*. *qGL-6* was localized on a 1.26 Mb region on chromosome 6, and *OsARF19* may be its candidate gene. Based on QTL mapping, three single-segment substitution lines (S1, S2, and S3) and two double-segment substitution lines (D1 and D2) were selected, and the mapping accuracy for *qGL-5* and *qGL-6* was further verified using three single-segment substitution lines. Analysis of QTL additive and epistatic effects revealed that the additive effect of alleles *qGL-5* and *qGL-6* from ‘Xihui 18’ was estimated to increase grain length of Z1392 by 0.22 and 0.15 mm, respectively. In addition, a positive epistatic interaction between *qGL-5* and *qGL-6* was detected, which indicates that the pyramiding of *qGL-5* and *qGL-6* for grain length produces a novel genotype with longer grains.

**Conclusions:**

Inheritance of grain length in the triple-segment substitution line Z1392 is mainly controlled by two major QTLs, *qGL-5* and *qGL-6*. *qGL-6* was found to be located in a 1.26 Mb region on chromosome 6, and *OsARF19* may be its candidate gene. A positive epistatic interaction between *qGL-5* and *qGL-6* results in longer grains. The present results can be used to facilitate cloning of the *qGL-5* and *qGL-6* genes and contribute to improvement of grain yield in rice.

## Background

Rice (*Oryza sativa* L.) is an important cereal crop throughout Asia. On the basis of the length: width ratio, rice grains are divided into three size classes, namely long, medium, and short grain, for which the ratios range from greater than 3.0, 2.1–2.9, and less than 2, respectively (Bai et al. 2010). Improvements in grain size are directly associated with yield, therefore grain size and yield-related traits are an important focus in research on rice. Chromosome segment substitution lines (CSSLs) are invaluable material in quantitative trait locus (QTL) research for the separation and localization of specific traits (Kubo and Yoshimura 2002). Quantitative traits, such as grain size, plant height, heading stage, and grain filling, are controlled by multiple genes. The construction of CSSLs has formed a strong foundation on which to base further research on quantitative traits.

Rice grain size is controlled by a combination of grain length, grain width, and grain thickness. Previous studies have shown that the development of grain size is dependent on multiple pathways. The mitogen-activated protein kinases (MAPK) pathway contains three cascade reactions, which play an important role in regulation of grain size (Li and Li 2016). *SMG1* encod es Mitogen Activated Protein Kinase Kinase 4 (OsMKK4), which is involved in the MAPK signaling pathway. The *smg1* mutant produces small and light grains due to a decreased cell number (Duan et al. 2014). *OsMAPK6* may be a downstream effector of OsMAKK4. Mutation of *OsMAPK6* also causes small-grain and dwarf phenotypes as a result of limited cell proliferation (Liu et al. 2015b). *GRAIN SIZE AND NUMBER 1* (*GSN1*) is a negative regulator of the *SMALL GRAIN 2* (*OsMKKK10*)*–SMALL GRAIN 1* (*OsMKK4*)*–OsMPK6* cascade reaction, and regulates cell differentiation and proliferation through the *GSN1–MAPK* pathway, thus regulating the number and length of grains (Guo et al. 2018). Guanine nucleotide-binding proteins (G proteins) consist of three subunits (α, β, and γ), and G protein-coupled receptors are also involved in the transduction of signaling pathways in rice (Liu et al. 2018). *qLGY3* encodes a variable splicing protein, OsMADS1^lgy3^, which is a crucial effector downstream of the G protein βγ dimer (Liu et al. 2018). The Gγ subunit *LONG KERNEL 3* (*GS3*) interacts with DENSE AND ERECT PANICLE 1 (DEP1) and MADS-domain transcription factors, and participates in the G-protein regulation pathway as a cofactor in the regulation of grain size. Concurrent expression of an allele of the grain size gene *OsMADS1*^*lgy3*^, *GS3*, and panicle *DEP1* may result in traits that enhance yield and quality (Liu et al. 2018). Several recent studies have demonstrated that the ubiquitin–proteasome pathway may also regulate the development of grain size in rice. *GRAIN WEIGHT 2* (*GW2*) encodes a RING-type protein with E3 ubiquitin ligase activity, which regulates proteolysis by targeting substrate binding to the proteasome, thereby negatively regulating cell proliferation and playing a role in the degradation of the ubiquitin–proteasome pathway. Loss of *GW2* increases cell number, which results in broader glumes and an accelerated rate of grain filling, thus increasing grain width, weight, and yield (Song et al. 2007). Epigenetic modification also regulates the development of grain size. *RELATED TO ABSCISIC ACID INSENSITIVE 3* (*ABI3*)*/VIVIPAROUS 1* (*VP1*) *6* (*RAV6*) encodes a B3 DNA-binding protein, which affects the brassinosteroid (BR) pathway by controlling the degree of promoter methylation, thereby regulating the leaf angle and grain size (Zhang et al. 2015b). *GW6a* encodes a novel *GANT*-like protein with histone acetyltransferase activity, which regulates rice grain size and yield by regulating the overall acetylation level of histone H4 (Song et al. 2015). In addition, many phytohormone-regulated pathways in rice control the proliferation and longitudinal growth of cells. Multiple genes are involved in the BR signaling pathway, for example, *DWARF EBISU* (*D2/SMG11*) encodes cytochrome P450, which regulates grain size by controlling cell elongation (Fang et al. 2016). Several genes are involved in auxin regulation, such as *BIG GRAIN 1* (*BG1*), which encodes a novel membrane-localized protein. Overexpression of *BG1* leads to a significant increase in grain size, with clearly perturbed gravitropism in severe cases, by changing the auxin basipetal transport and altered auxin distribution; this suggests that *BG1* plays a role in the auxin regulatory pathway (Liu et al. 2015a). Genes involved in the cytokinin regulatory pathway, such as *REGULATOR OF AWN ELONGATION 2* (*GAD1*), which encodes an epidermal pattern factor, regulate the content of endogenous cytokinins by interacting with GRAIN NUMBER 1A (OsCKX2) and DROUGHT AND SALT TOLERANCE (DST) to regulate grain size (Jin et al. 2016). In general, many of the modes that regulate rice grain size remain unexplained and require further study, owing to the complex regulatory modes within and among the pathways.

In this study, a novel rice CSSL with long grains Z1392 and carrying three substitution segments was derived from a cross between ‘Nipponbare’ as the recipient parent and the *indica* restorer line ‘Xihui 18’ as the donor parent. We performed QTL mapping of grain size traits using a secondary F_2_ population derived from the cross between ‘Nipponbare’ and Z1392. *qGL-6* was localized on a 1.26 Mb region on chromosome 6, and *OsARF19* may be its candidate gene. On the basis of the QTL mapping results, we selected single-segment (SSSL), double-segment (DSSL), and triple-segment substitution lines (TSSL) for each QTL in the F_3_ generation using marker-assisted selection (MAS). We also carried out analysis of the additive and epistatic effects of QTLs on grain length. The results in the present study will be helpful in cloning of QTLs for grain length and their breeding application in the future.

## Results

### Identification of Substitution Segments in Z1392

**Fig. 1:**
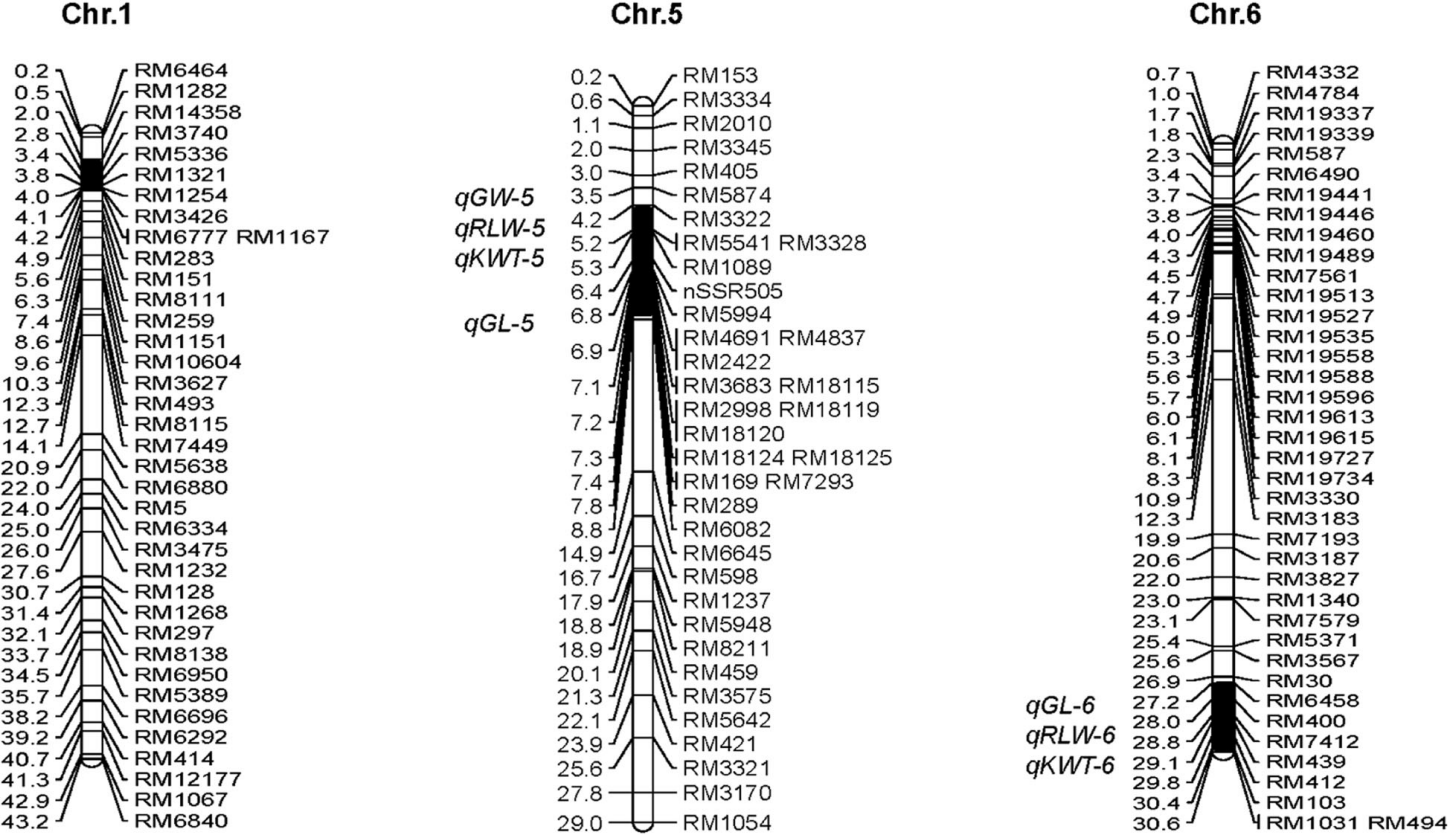
Substitution segments harbored in the CSSL Z1392 and position of grain size related QTLs. Physical distance (Mb) is specified on the left of each chromosome (based on the Nipponbare reference genome) and markers are specified on the right. The solid black segment is the substitution fragment region from the donor Xihui 18 and the identified QTLs are listed on the left of each chromosome in italics. *qGL*, QTL for grain length; *qGW*, QTL for grain width; *qRLW*, QTL for ratio of length to width; *qKWT*, QTL for 1000-grain weight

Three substitution segments of Z1392 originating in Xihui 18 were located on chromosomes 1, 5, and 6. The substitution segment on chromosome 1 was the the short arm--RM3426--RM1167 and its estimated length was 2.1 Mb. The substitution segment on chromosome 5 was RM3345--nSSR505–RM18119-RM289--RM6082 and it had an estimated length of 3.9 Mb. The substitution segment on chromosome 6 was RM5371--RM7412–RM494--long arm and its estimated length was 3.5 Mb. The total substitution length was 9.5 Mb and the mean length was 3.17 Mb (Fig. 1).

### Grain Size Related Traits Analysis of Z1392

**Fig. 2:**
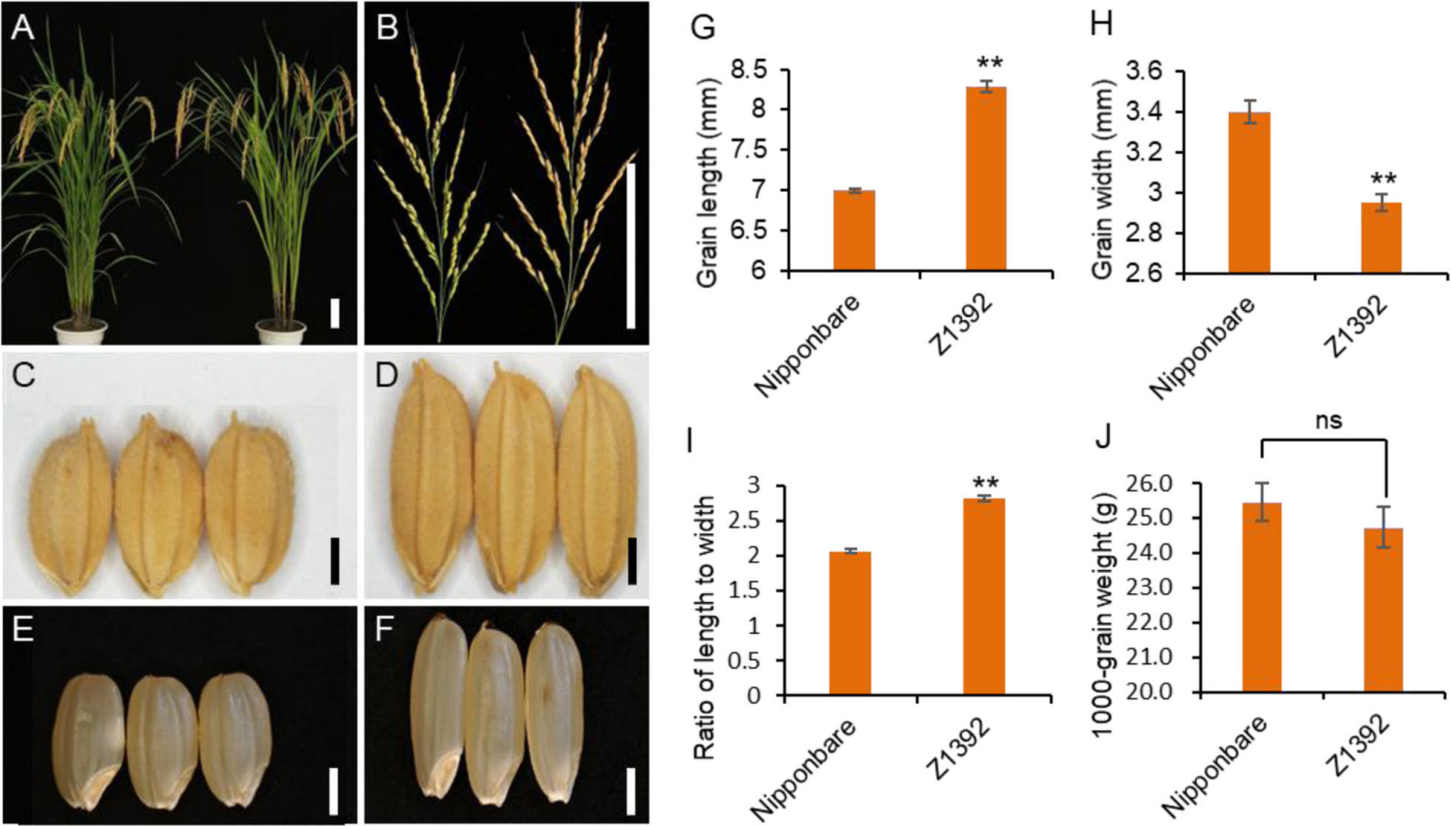
Phenotype of Nipponbare and Z1392. **a**, Plant type of Nipponbare (left) and Z1392 (right). **b**, Main panicle of Nipponbare (left) and Z1392 (right). **c**, **d**, Grains of Nipponbare (**c**) and Z1392 (**d**). **e**, **f**, Brown grains of Nipponbare (**e**) and Z1392 (**f**). **g**-**j**, Grain length (**g**), grain width (**h**), ratia of length to width(**i**) and 1000-grain weigth(**j**) of Nipponbare and Z1392. Bars in A and B, 10 cm; C–F, 2 mm

Z1392 has similar plant type with the recipient Nipponbare (Fig. 2 A, B). However, Compared with Nipponbare, Z1392 showed a grain length that was increased by 18.43%, a grain width decreased by 13.49% and a ratio of length to width increased by 36.41% (*p* < 0.01 for each trait) (Fig. 2 C-I). There was no difference for 1000-grain weight between Z1392 and Nipponbare (Fig. 2j).

### Cytological Analysis of Glumes in Z1392 and Nipponbare

**Fig. 3:**
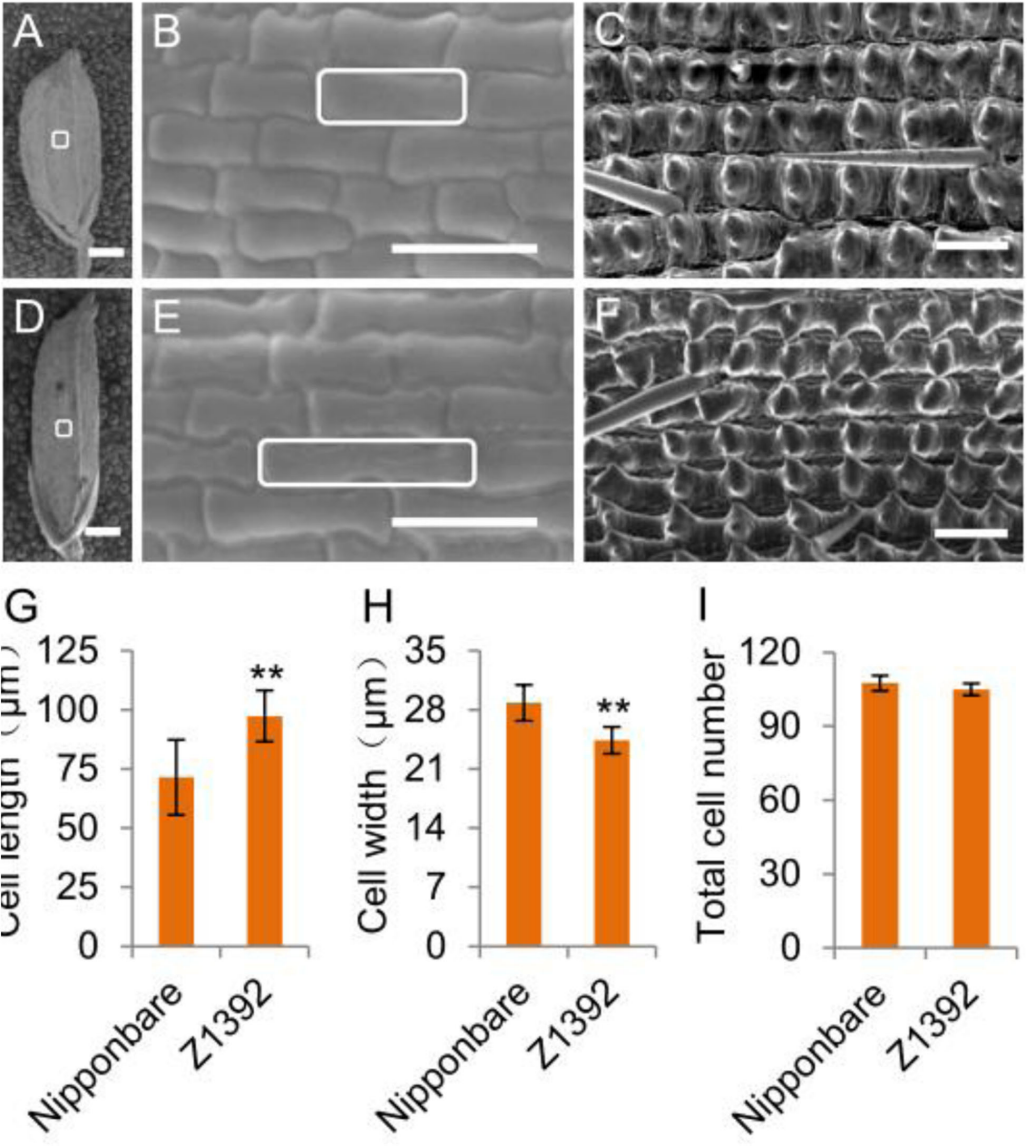
Scanning electron microscopic observation and analysis of the glume. **a**–**c**, Scanning electron micrograph of the lemma (**a**, **d**), and inner epidermis (**b**, **e**) and outer epidermis (**c**, **f**) of the glume of Nipponbare (**a**-**c**) and Z1392 (**d**-**f**). **g**-**h**, Cell length and cell width in the inner epidermis of the lemma of Nipponbare and Z1392. **i**, Total cell number in the outer epidermis of the lemma along the longitudinal axis of Nipponbare and Z1392. Bars in A and B, 1 mm; B, C, E and F, 100 μm. * and ** indicate a significant difference between the two parents at *P* < 0.05 and *P* < 0.01, respectively

To examine the factors responsible for the increase in grain length of the substitution line Z1392, scanning electron microscopy was used to observe the cell morphology of glumes in Nipponbare and Z1392 at the heading stage. We measured cell size in the inner epidermis of the glumes of mature grains. The glume cell length in Z1932 was 25.93 μm longer than that of Nipponbare, whereas the cell width was narrower by 4.42 μm on average (Fig. 3a, b, d, e, g, h). No significant difference in total cell number in the outer epidermis of the glume along the longitudinal axis was observed between Nipponbare and Z1392 (Fig. 3c, f, i). These findings suggest that the increase in grain length of Z1392 might have resulted predominantly from cell expansion, and not from an increase in cell number.

### Genetic Analysis of Grain Size Related Traits of Z1392

**Fig. 4:**
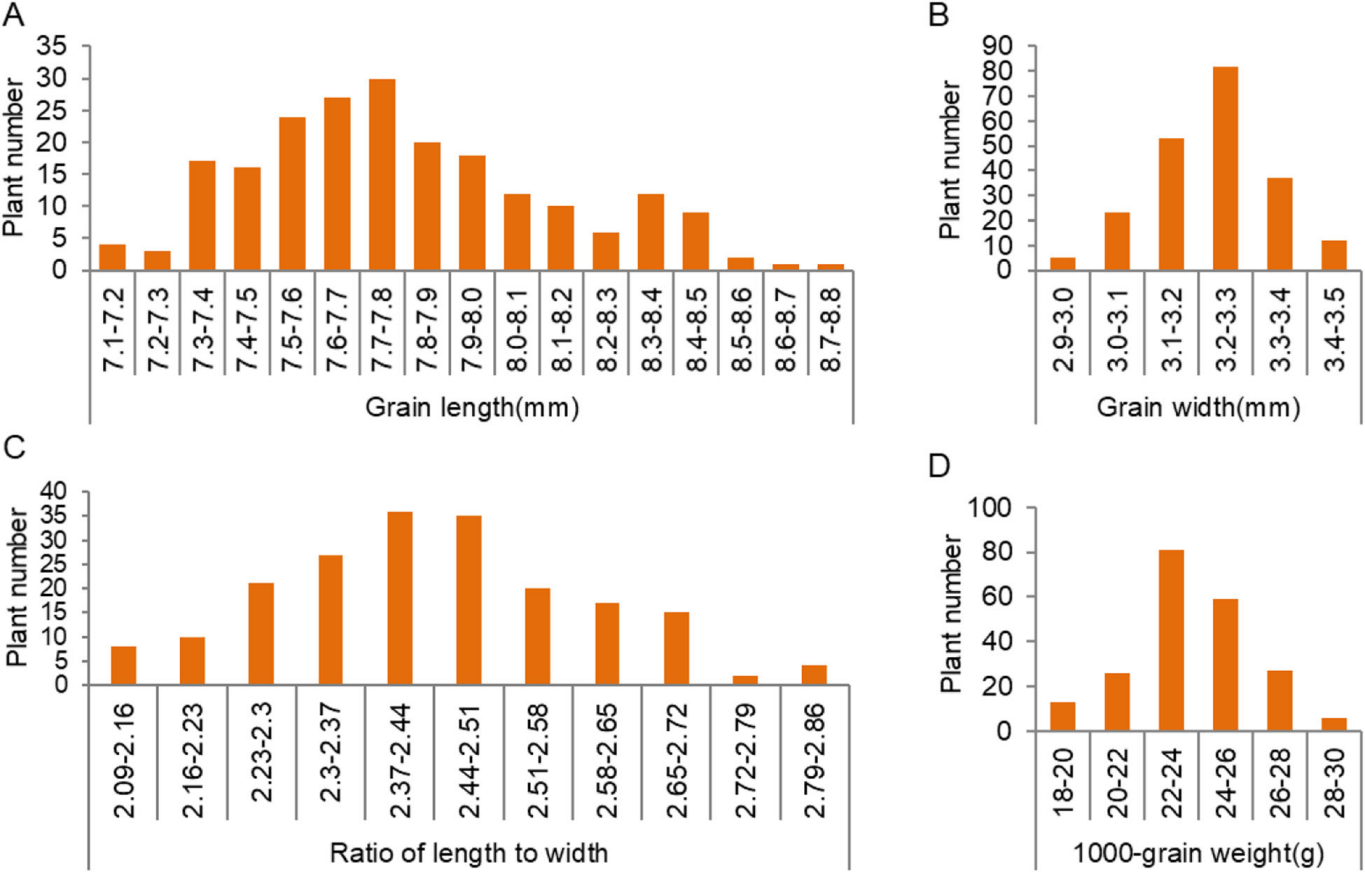
Frequency distributions of grain size related traits in the F_2_ population

The grain size in Nipponbare displayed a short and broad phenotype, whereas the grain was long and narrow in Xihui 18 and Z1392. The grain size in F_1_ individuals derived from the cross between Nipponbare and Z1392 was long and narrow, which indicates that the long grain trait exhibited dominance over the short grain phenotype. In the F_2_ population of 216 individuals, the traits related to grain size, including grain length, grain width, ratio of length to width and 1000-grain weight showed basically a normal distribution, which indicates that these traits in Z1392 were still controlled by multiple genes (Fig. 4).

### QTL Mapping for Grain Size Related Traits in the Secondary F_2_ Population of Nipponbare /Z1392

Seven quantitative trait loci for grain size related traits were identified in the secondary F_2_ population obtained from the cross between Nipponbare and Z1392. The inheritance of grain length in Z1392 was controlled by two major QTLs, *qGL-5* and *qGL-6*. The additive effect of alleles of *qGL-5* and *qGL-6* inherited from Xihui 18 was estimated to increase the grain length by 0.22 mm and 0.15 mm in Z1392, implying contribution rates of 32.25% and 15.07%, respectively. The grain width of Z1392 was controlled by the negative effect of *qGW-5*, which could reduce grain width in Z1392 by 0.09 mm per grain, and its contribution rate was 73.38%. Similarly, the ratio of length to width of Z1392 was controlled by two QTLs, *qRLW-5* and *qRLW-6*, and the contribution rates were 19.01% and 13.52%, respectively. Two QTLs (*qKWT-5* and *qKWT-6*) showed negative and positive effects on 1000-grain weight, and the contribution rates were 20.12% and 21.81%, respectively (Table 1).

**Table 1:** QTL mapping for grain size related traits in the secondary F_2_ population of Nipponbare /Z1392

Traits	QTL	Chr.	Linked marker	Estimated effect ± SE	Var%	***P***-value
Grain length	*qGL-5*	5	RM18119	0.22 ± 0.07	32.25	0.0012
Grain length	*qGL-6*	6	RM7412	0.15 ± 0.07	15.07	0.0282
Grain width	*qGW-5*	5	nSSR505	−0.09 ± 0.02	73.38	< 0.0001
Ratio of length to width	*qRLW-5*	5	nSSR505	0.06 ± 0.02	19.01	0.0016
Ratio of length to width	*qRLW-6*	6	RM7412	0.05 ± 0.02	13.52	0.0278
1000-grain weight	*qKWT-5*	5	nSSR505	−1.08 ± 0.33	20.12	0.0015
1000-grain weight	*qKWT-6*	6	RM7412	1.12 ± 0.42	21.81	0.0075

### Fine Mapping of Putative *qGL-6* and Sequence Analysis of Candidate Genes

**Fig. 5:**
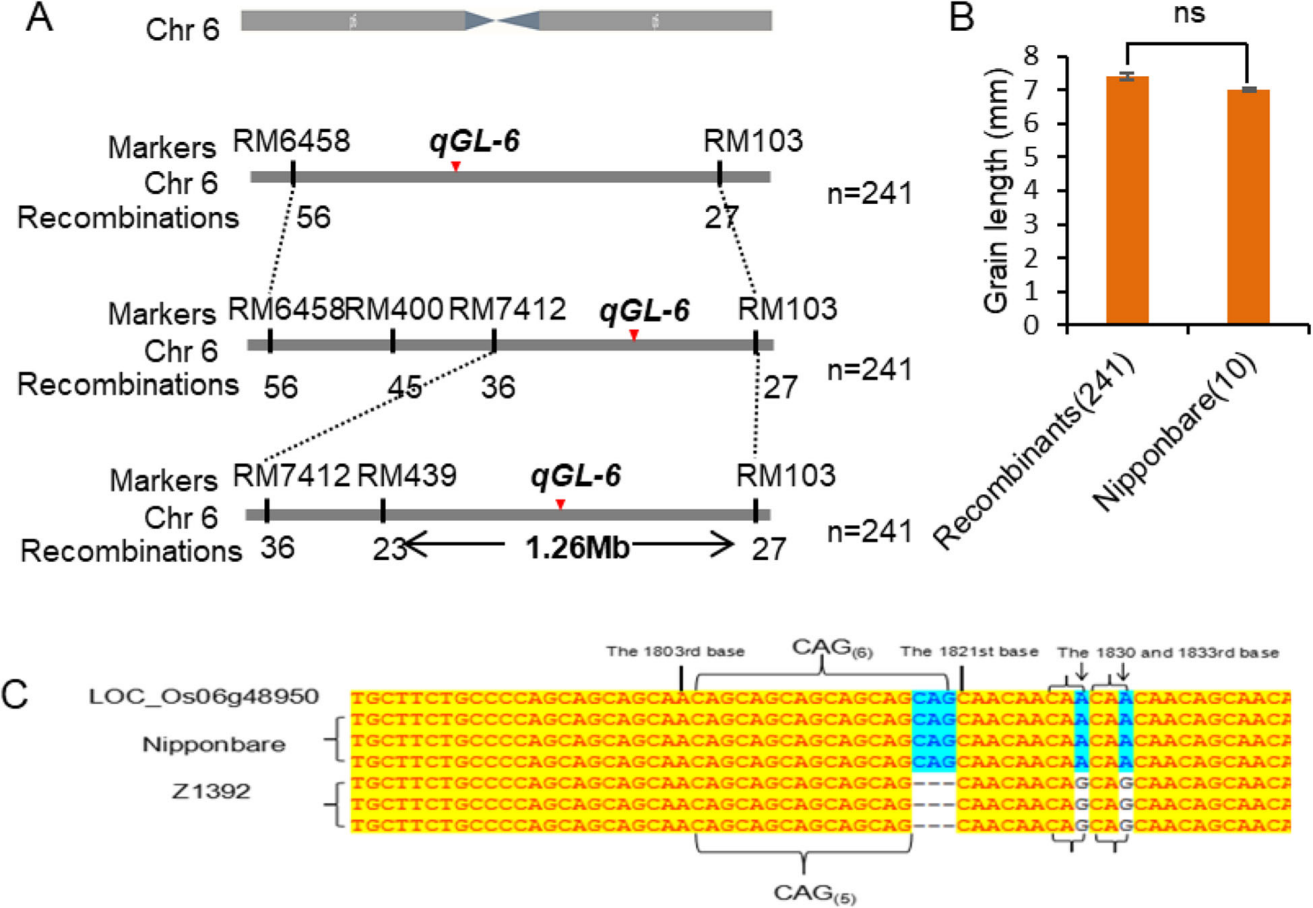
Fine mapping of putative *qGL-6* and sequence analysis of candidate genes. **a***qGL-6* was mapped to an interval of 1.26 Mb. **b** The grain length of the 241 recombinants and 10 Nipponbare. **c** The DNA sequence of *OsARF19* in Z1392 compared with Nipponbare

On the basis of QTL mapping, 241 recessive individuals with short grains excluding those with bands of Nipponbare in the *qGL-5* locus of the F_2_ population were used for fine mapping of *qGL-6*. The grain length was 7.40 mm in the 241 recombinant types, which shows no real difference from that (7.0 mm) seen in Nipponbare (Fig. 5b). This enabled *qGL-6* to be fine-mapped between RM439 and RM103 on chromosome 6, with a physical distance of 1.26 Mb (Fig. 5a). Through gene prediction and sequencing, the auxin response factor *OsARF19* was identified as a candidate gene of *qGL-6*, and this highlighted a number of differences in the DNA sequence of *OsARF19* between Nipponbare and Z1392. Firstly, there are 6 CAGs in the CAG repeat region after the 1803rd bases in Nipponbare, but only 5 CAGs in Z1392, and the CAG encodes glutamine. Thus, a glutamine of the *OsARF19* protein in Z1392 is reduced compared to Nipponbare. Secondly, a base in the 1830th and 1833rd bases in Nipponbare was changed to G base in Z1392. Thus the CAA in Nipponbare was changed to the CAG in Z1392, but both of these encoded glutamine, and did not cause amino acid changes (Fig. 5c).

### Analysis of Additive and Epistatic Effects of QTLs on Grain Length

**Fig. 6:**
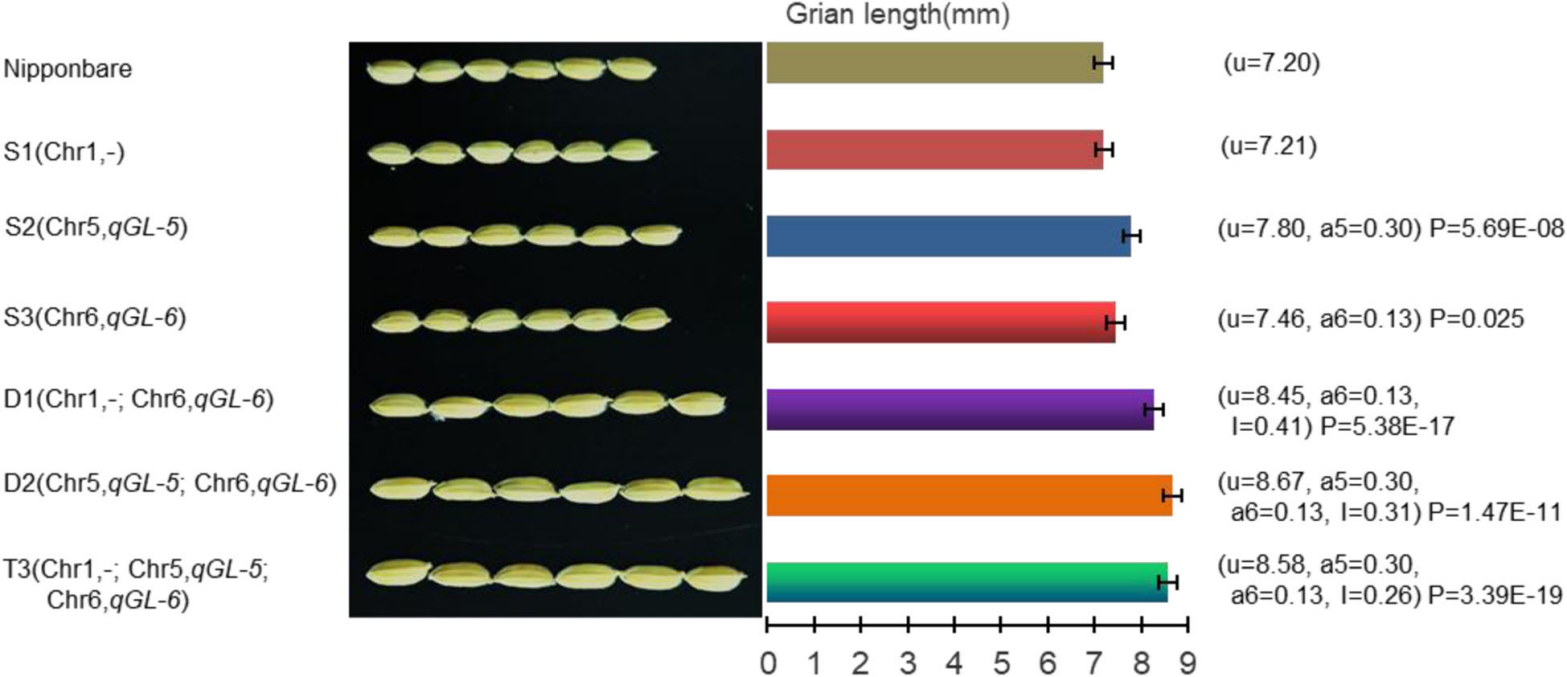
Phenotype and genotype analysis of six chromosome segment substitution lines. u is the phenotypic value; a denotes the additive effect; I denotes the epistatic effect. A *t*-test was used to analyse significant difference

On the basis of the QTL mapping results, eight individuals were selected from the F_2_ population by MAS, and F_3_ lines were obtained from each individual. Ten individuals for each F_3_ line were sampled for further molecular marker selection using heterozygous markers in the selected plant lines. Ultimately, three SSSLs (S1, S2, and S3) and two DSSLs (D1 and D2) were selected. S1 carried the substitution fragment of chromosome 1 and lacked the QTLs for grain length and grain width. S2 carried the substitution fragment of chromosome 5, which included the grain length QTL *qGL-5*, for which the additive effect was estimated to be an increased grain length of S2 by 0.3 mm. S3 carried the chromosome 6 substitution fragment containing the grain length QTL *qGL-6*, for which the additive effect was estimated to be an increased grain length of S3 by 0.13 mm (Fig. 6). These QTLs were repeatedly detected in different years (*qGL-5*: a_2017_ = 0.22, *P* = 0.0012; a_2018_ = 0.22, *P* = 0.0012; *qGL-6*: a_2017_ = 0.15, *P* = 0.028; a_2018_ = 0.22, *P* = 0.0012), which indicates that the QTLs were mapped accurately, and differences in the additive effect of the QTLs were detected in comparison with the previous season, which suggests that different environments have interactive effects on grain length.

The DSSL D1 carried the substitution fragments of chromosomes 1 and 6. The substitution fragment of chromosome 1 lacked a grain length QTL, whereas the substitution fragment of chromosome 6 contained *qGL-6*. The additive effect of *qGL-6* was 0.13 mm, and an epistatic effect between the two substitution segments of 0.41 mm was observed. The DSSL D2 carried substitution fragments of chromosomes 5 and 6. The substitution fragment of chromosome 5 contained *qGL-5*, for which the additive effect was 0.3 mm, the substitution fragment of chromosome 6 contained *qGL-6*, which showed an additive effect of 0.13 mm, and an epistatic effect between the two substitution fragments of 0.31 mm was observed. The TSSL T3 (Z1392) carried the three substitution fragments. As indicated in the preceding discussion, the substitution fragment of chromosome 1 lacked grain length QTL, whereas those of chromosomes 5 and 6 contained *qGL-5* and *qGL-6*, respectively. An epistatic effect among the three substitution fragments of 0.26 mm was observed. Therefore, the theoretical genetic effects on grain length of D1, D2, and T3 were 0.44 mm, 0.74 mm, and 0.69 mm, respectively. Given that the grain length of the recipient parent Nipponbare was 7.2 mm, the grain length of D1, D2, and T3 was predicted to be 8.15 mm, 8.29 mm, and 8.24 mm, respectively. However, the actual average grain length of D1, D2, and T3 was 8.45 mm, 8.67 mm, and 8.58 mm, which may be the result of errors or environmental effects between individuals (Fig. 6). Taken together, the combination of *qGL-5* and *qGL-6* resulted in the development of a longer grain.

## Discussion

In this study, the rice CSSL Z1392 was identified, having been derived from a cross between Nipponbare as the recipient parent and the *indica* restorer line Xihui 18 as the donor parent. Z1392 carried three substitution segments and exhibited a long-grain phenotype. The major QTLs carried by Z1392, *qGL-5* and *qGL-6*, contributed to grain length, which affords the opportunity for future research on the molecular mechanisms underlying the development and regulation of grain length. In addition, we analyzed two SSSLs that carried *qGL-5* or *qGL-6*, which positively affected grain length, and one DSSL that carried *qGL-5* and *qGL-6*. Interaction of *qGL-5* and *qGL-6* resulted in positive epistatic effects in the DSSL, with longer grain length compared with the single long-grain SSSL. Thus, combination of major QTLs that positively affect grain length resulted in a further increase in grain length. However, a previous study showed that combination of two QTLs, *qGL3* and *qGL4-b*, which each have positive effects on grain length, resulted in negative epistatic effects in the DSSL and thus did not result in a longer grain (Zhao et al. 2011). Nevertheless, these results are not contradictory, but rather verify the finding that QTLs with different effects on grain length will produce different interactive effects when combined, and only when the epistatic effect is in the same direction as the additive effect of the target gene can any significant improvement in grain length be achieved. To achieve the desired effect of the target gene, it is first necessary to predict whether two genes interact with each other, and the effect of that interaction. Therefore, given that Z1392 harbors *qGL-5* and *qGL-6*, this line is an important resource for molecular breeding of rice.

Senven QTLs for grain size related traits were identified in Z1392. Compared with the reported QTLs, the grain size QTLs *qGW-5* and *qKWT-5* are located in the same chromosomal region as *GW5*, which participates in the ubiquitin–proteasome pathway to regulate cell division during seed development (Weng et al. 2008). The grain length QTL *qGL-6* was fine-mapped on chromosome 6, with a physical distance of 1.26 Mb, and 3-bp Indel (CAG) occurred in the coding region of *OsARF19*, which encodes an auxin response factor. The null mutant *osarf19* (isolated from a T-DNA) and RNAi lines of *OsARF19* displayed enlarged organs and plant architecture caused by cell elongation (Zhang et al. 2015a, 2015b). Similarly, mutation of *qGL-6* showing increased grain length and plant height in Z1392 might also result predominantly from cell expansion. We therefore suppose that *OsARF19* is the candidate for *qGL-6*. The localization interval for the grain length QTL *qGL-5* may contain five genes associated with the development of grain size, and which encode a Serine/Threonine protein phosphatase, a rapid alkalization factor (RALF) family protein precursor, a GSK3/SHAGGY-Like kinase, a protein kinase, and an expressed protein containing a PPR repeat sequence. Although these genes that control different traits are located in the same chromosomal interval as the QTLs identified in the present study, further sequencing and functional complementation are still needed to determine whether the genes are alleles of the identified QTLs. Therefore, the present results lay the foundations for additional fine localization of the QTLs, cloning of the candidate genes, and functional research on grain size.

## Conclusions

The rice TSSL line Z1392 was identified, which exhibited increased grain length. The chromosomal substitution fragments were located on chromosomes 1, 5, and 6, and the average substitution length was 3.17 Mb. The increased grain length in Z1392 might be caused predominantly by cell expansion in the glumes. Inheritance of the long-grain phenotype in Z1392 is mainly controlled by two major QTLs, *qGL-5* and *qGL-6*. *qGL-6* was localized to a 1.26 Mb region on chromosome 6, and *OsARF19* may be its candidate gene. On the basis of QTL mapping, three SSSLs and two DSSLs were selected. Epistatic effect analysis revealed a positive epistatic interaction between *qGL-5* and *qGL-6*, which indicates that pyramiding of *qGL-5* and *qGL-6* enhances the long-grain phenotype.

## Materials and Methods

### Plant Materials

The rice CSSL Z1392 was developed using the rice cultivars ‘Nipponbare’ as the recipient parent and excellent indica restore line ‘Xihui 18’ as the donor parent. After continuous backcrossing and selfing, in combination with phenotype-based selection and simple sequence repeat (SSR) marker selection, a genetically stable CSSL with three substitution segments was identified and designated Z1392.

The plant material used for QTL mapping was a secondary F_2_ population derived from a cross between Nipponbare and Z1392.

On the basis of QTL mapping results obtained in 2017, eight individual plants were selected by MAS and planted in 2018. Ten individual plants were selected from each line to allow further selection of molecular markers for hybrid markers, and homozygous single-fragment substitution lines and double-fragment substitution lines were then selected.

### Material Planting Method

In June 2016, the F_1_ was generated at the experimental station of Southwest University in Chongqing, China, by crossing Nipponbare with Z1392. In August, hybrid seeds were planted in LingShui, Hainan Province. All 30 F_1_ seeds and seeds of the parents were planted at the experimental station of Southwest University in Chongqing on March 10, 2017. In March 2018, on the basis of QTL mapping results in 2017, 30 seeds of eight individual plants were used for breeding of secondary substitution fragment lines, and the parents were planted in the same experimental field. On April 15, 2018, all plants were transplanted to the same experimental field. The spacing between hills was 16.67 cm and the spacing between rows was 26.67 cm. Conventional field management practices were applied.

### Identification of Substitution Segments in Z1392

A set of 263 markers polymorphic between Nipponbare and Xihui 18 were selected from 429 markers that covered the entire rice genome. The long-grain substitution line Z1392, harboring three substitution segments, was selected from the BC_3_F_4_ generation by selection of molecular marker and phenotype. The identification of substitution segments was performed as described previously (Zhao et al. 2016), and the estimated length of the substitution segments was calculated following an established method (Paterson et al. 1991). The distance of the substitution markers from the donor plus half of the distance between the boundary markers from Nipponbare and the substitution markers was taken to be the estimated substitution length. Mapchart 2.2 was used to draw a chromosome substitution fragment map.

### Grain Size Related Traits Assessment

At maturity, 10 plants on the third to seventh hills of the central two rows of the Nipponbare and Z1392 plots, and 10 plants of selected SSSLs and DSSLs, and 216 individuals were harvested for QTL mapping. For each plant, grain length, grain width, ratio of length to width, and 1000-grain weight, were measured. A Student’s *t*-test was conducted for each trait to assess the significance of differences between Nipponbare and Z1392, and descriptive statistics such as skewness and kurtosis were obtained for the F_2_ population using the statistical functions in Microsoft Excel 2010.

### Scanning Electron Microscopy

At the completion of the booting stage and before the heading period, the phenotypic characteristics of the inner and outer epidermal cells of the glume in Nipponbare and Z1392 were investigated using a Hitachi SU3500 scanning electron microscope (Hitachi, Tokyo, Japan) with a frozen stage (− 40 °C) under a low-vacuum environment.

### QTL Mapping

Total genomic DNA of Nipponbare, Xihui 18, Z1392, and the 216 plants from the F_2_ population was extracted using the cetyltrimethylammonium bromide method (Mccouch et al. 1988). PCR amplification, non-denaturing polyacrylamide gel electrophoresis, and rapid silver staining were performed as described previously (Zhao et al. 2016). Nipponbare bands were scored as “− 1”, Z1392 bands were scored as “1”, heterozygous bands were scored as “0”, and the absence of marker bands was scored as “.”. The marker assignments of all six SSR markers on the substitution segments of Z1392, together with the phenotypic values of each individual in the F_2_ population, were used for QTL mapping. QTL mapping was performed using the restricted maximum likelihood method by mixed linear models (MLM) implemented in the HPMIXED procedure of SAS (SAS Institute Inc., Cary, NC, USA), with significance determined at α = 0.05 (Hu and Xu 2009; Cui et al. 2017; Spilke et al. 2005).

### Development of SSSLs and DSSLs, and Additive and Epistatic Effect Analysis

On the basis of the QTL mapping results obtained in 2017, eight plants were selected using a MAS method and were planted in 2018. Ten plants from each line were selected for further molecular marker assisted selection. Ultimately, homozygous SSSLs and DSSLs were selected. At the maturity stage, 10 plants from each SSSL, DSSL, and TSSL (Z1392) were sampled and the grain length and grain width of each plant were measured, with three replicate measurements per plant recorded. Additive and epistasis effects of unlinked QTLs for grain length and grain width were calculated following the descriptions of Eshed and Zamir (Eshed and Zamir 1996). The additive effect was taken as half the difference between each SSSL and the recipient Nipponbare at *P* < 0.01 according to the student *t*-test. QTL interaction in each DSSL was determined by comparing the difference between the effect of DSSL and its corresponding SSSL pairs (SSSLa and SSSLb) at *P* < 0.01. QTL interaction in TSSL was determined by comparing the difference between the effect of (TSSL+ Nipponbare +Nipponbare) and its corresponding SSSL pairs (SSSLa+ SSSLb + SSSLc) at *P* < 0.01 by student *t*-test. The epistatic effect in DSSL was estimated using phenotypic values according to the value of half [DSPL + Nipponbare) - (SSSLa + SSSLb)]. The epistatic effect in TSSL was estimated using phenotypic values according to the value of half [(TSSL + Nipponbare+ Nipponbare) - (SSSLa + SSSLb+ SSSLc)]. Then, a *t-*test was used to validate QTLs for all traits between each SSSL and Nipponbare. The additive effect of a QTL is half the difference between each SSSL and Nipponbare, and the threshold probability value for a QTL is less than 0.05 (Eshed and Zamir 1996).

## Supplementary information

**Additional file 1.** Supplemental Table 1. Primers used in the study.

## Data Availability

The datasets supporting the conclusions of this article are included within the article.

## Abbreviations

QTL: Quantitative trait loci
SSSL: Single-segment
CSSLs: Chromosome segment substitution lines
DSSL: Double-segment
TSSL: Triple-segment substitution lines
SSR: Simple sequence repeat
SEM: Scanning electron microscopy
MAS: Marker-assisted selection
