# Supplementary material for: Analysis of QTL for Grain Size in a Rice Chromosome Segment Substitution Line Z1392 with Long Grains and Fine Mapping of qGL-6
Source: Rice (N Y). 2020 Jun 11;13:40. doi: 10.1186/s12284-020-00399-z (PMC7290020; doi:10.1186/s12284-020-00399-z)
Supplement: Supplementary file 1 — Additional file 1. Supplemental Table 1. Primers used in the study. [file 12284_2020_399_MOESM1_ESM.docx]

| **Supplemental Table 1. Primers used in the study.** | | |  |
| --- | --- | --- | --- |
| Purpose | Primer name | Sequence | Remarks |
| Mapping | RM6458-F | TTGTCACGAGAGATGTGAGAGTGAGC | SSR |
|  | RM6458-R | GGGTCTTCGAGGATGGAGTTGG | SSR |
|  | RM400-F | TTACACCAGGCTACCCAAACTCG | SSR |
|  | RM400-R | TTGCTGAGTTCCCTCGTCTATCC | SSR |
|  | RM7412-F | CGAGTGGATCAGCAAATCTACAGC | SSR |
|  | RM7412-R | CAGCATCAGGCTTGTGTTAATGG | SSR |
|  | RM439-F | CTGGGTCTAATCTCGTCCTAAATTGC | SSR |
|  | RM439-R | CGCCTCTCATAACAGTCCACTCC | SSR |
|  | RM103-F  RM103-R | ATCAGCAGCATTCAGCATTTGG  CCGGACGATGTGTATATCTCTTGG | SSR |
|  |  |  | SSR |
| Sequencing | OSARF19-1-F | ACTTCATCCTGCAATTCTACCTCAC |  |
|  | OSARF19-1-R | CCAAAGGGTACTCGATCACGATG |  |
|  | OSARF19-2-F | CGTGTGATGGCTATGTCCTCATAG |  |
|  | OSARF19-2-R | CTGCTGTTGCATATCCAGAAGTAACT |  |
|  | OSARF19-3-F | GAGTAAGATGCCTGCACAGTTGTC |  |
|  | OSARF19-3-R | GACAATGCTTGCTGACACCTATGT |  |
